# Tiny but Mighty: *Vipera ammodytes meridionalis* (Eastern Long-Nosed Viper) Ontogenetic Venom Variations in Procoagulant Potency and the Impact on Antivenom Efficacies

**DOI:** 10.3390/toxins16090396

**Published:** 2024-09-14

**Authors:** Zichen Qiao, Lee Jones, Lachlan A. Bourke, Lorenzo Seneci, Abhinandan Chowdhury, Aude Violette, Rudy Fourmy, Raul Soria, Matt Aldridge, Bryan G. Fry

**Affiliations:** 1 Adaptive Biotoxicology Lab, School of the Environment, University of Queensland, St. Lucia, QLD 4072, Australia; zichen.qiao@uq.net.au (Z.Q.); lee.jones1@uqconnect.edu.au (L.J.); l.bourke@uq.net.au (L.A.B.); uqlsenec@uq.edu.au (L.S.); abhinandan.choudhury@uq.edu.au (A.C.); 2 Alphabiotoxine Laboratory Sprl, Barberie 15, 7911 Montroeul-au-Bois, Belgium; aude.violette@alphabiotoxine.com (A.V.); info@alphabiotoxine.be (R.F.); 3 Inosan Biopharma, 28108 Alcobendas, Madrid, Spain; rsoria@inosanbiopharma.com; 4 MicroPharm Limited, Newcastle Emlyn SA38 9BY, UK; matt.aldridge@micropharm.co.uk

**Keywords:** Echis, coagulopathy, antivenom, small-molecule enzyme inhibitor, factor activation

## Abstract

The Eastern Long-Nosed Viper (*Vipera ammodytes meridionalis*) is considered one of the most venomous snakes in Europe. However, it is unknown whether ontogenetic variation in venom effects occurs in this subspecies and how this may impact antivenom efficacy. In this study, we compared the procoagulant activities of *V. a. meridionalis* venom on human plasma between neonate and adult venom phenotypes. We also examined the efficacy of three antivenoms—Viperfav, ViperaTAb, and Inoserp Europe—across our neonate and adult venom samples. While both neonate and adult *V. a. meridionalis* venoms produced procoagulant effects, the effects produced by neonate venom were more potent. Consistent with this, neonate venom was a stronger activator of blood-clotting zymogens, converting them into their active forms, with a rank order of Factor X >> Factor VII > Factor XII. Conversely, the less potent adult venom had a rank order of FXII marginally more activated than Factor VII, and both much more so than Factor X. This adds to the growing body of evidence that activation of factors besides FII (prothrombin) and FX are significant variables in reptile venom-induced coagulopathy. Although all three examined antivenoms displayed effective neutralization of both neonate and adult *V. a. meridionalis* venoms, they generally showed higher efficacy on adult venom than on neonate venom. The ranking of antivenom efficacy against neonate venom, from the most effective to the least effective, were Viperfav, Inoserp Europe, ViperaTAb; for adult venom, the ranking was Inoserp Europe, Viperfav, ViperaTAb. Our data reveal ontogenetic variation in *V. a meridionalis*, but this difference may not be of clinical concern as antivenom was effective at neutralizing both adult and neonate venom phenotypes. Regardless, our results highlight a previously undocumented ontogenetic shift, likely driven by the documented difference in prey preference observed for this species across age classes

## 1. Introduction

Snakebite is a globally neglected disease and an important public health problem [1]. It is estimated that up to 5.5 million snakebites, 1.8 million envenomings, and 94,000 human deaths occur annually [1]. These numbers are recognized as gross underestimations because of poor/non-existent epidemiological records kept in some of the most affected regions [2,3]. Effects from snakebite are often systemic, but many long-term sequelae occur due to severe local effects, such as local necrosis leading to amputation [4,5]. Snake venoms are cocktails of components that collectively take action in prey capture, digestion, and self-defense [6]. Some components in snake venom attack the hemostatic system of victims, resulting in disruption of blood clotting through either an anticoagulant or procoagulant mechanism.

Procoagulant venoms activate the zymogen form of blood-clotting enzymes, leading to the generation of endogenous thrombin, which, in turn, converts fibrinogen into fibrin clots [7,8,9,10,11,12,13,14,15,16,17,18,19,20,21,22,23,24,25,26,27,28]. Anticoagulant venoms cause hemorrhagic shock in prey and human snakebite victims, while procoagulant venoms induce stroke in prey but lead to consumptive coagulopathy in the larger blood volume of human victims [5,29,30]. In contrast to the intense research on the anticoagulant mechanism, procoagulant toxicity has received comparably less attention, and of these efforts, the major focal area has been on the activation of Factor X and prothrombin, with the activation of other factors comparably neglected.

Although some venom components are shared between different snake lineages [31,32], extensive interspecific venom variation exists [33,34,35]. Even within the same species, venom variation can occur between sexes, age groups, and regional populations [12,16,25,26,36,37,38,39,40,41,42,43]. Such variations within a species can have profound impacts on antivenom efficacy, leading to poor outcomes [7,8,9,11,13,19,20,25,26,39,40,44,45,46,47,48,49,50,51,52,53,54,55,56,57,58,59,60,61,62].

Snake venoms are made up of proteinaceous toxins, many of which are dynamic, displaying accelerated rates of duplication and diversification [63]. Variations in the surface biogeographic features of toxins can strongly influence antivenom recognition, even between toxins that do not vary in functional sites, leading to potential clinical issues [20,46,49,50,51,61,62]. Examples include *Causus* species, which cleave fibrinogen in a destructive manner, with the venom of the short-glanded species *C. lichtensteinii* not neutralized by the South African SAIMR antivenom but the venom of the long-glanded species *C. maculatus* neutralized at the same antivenom:venom ratio [64]; *Crotalus scutulatus* subspecies, which produce flaccid paralysis through a presynaptic action have some subspecies well neutralized, but others not [39]; and *Trimeresurus* species, which have extreme variation in the ability of antivenom to neutralize their pathophysiological cleavage of fibrinogen to form weak, transient fibrin clots in a pseudo-procoagulant manner. In this study, the best and worst antivenom-neutralized species were each other’s closest relatives, while the second-best neutralized species was distant [53]. This study reinforces the paradigm that organismal relationships are poor predictors of antivenom efficacy.

In other cases, fundamental differences in the underlying venom biochemistry lead to highly variant antivenom effects. As an example, *Bothrops atrox* adult venoms were shown to vary widely between different Brazilian populations in their ability to activate FX versus FII (prothrombin) [22]. As the antivenom was made using a population rich in FII (prothrombin)-activating toxin while containing less FX-activating toxin, it was shown that populations with venom rich in FX-activating toxin were poorly neutralized.

Diet is considered the predominant driving force of venom variation [23,65,66,67,68,69,70]. Juveniles of some species are known to consume different prey types and utilize different foraging strategies and prey-handling behaviors compared to adult snakes [33]. For example, there is an extraordinary venom variation within the genus *Pseudocerastes.* While *Pseudocerastes fieldi* venom is potently neurotoxic [71,72,73,74,75], conversely, *Pseudocerastes urarachnoides* venom is strongly procoagulant, being a powerful activator of Factor X and prothrombin [17]. In such cases, shifts in venom components at different ages are hypothesized to improve effective prey immobilization. For some species, proteomic variations in venom biochemistry were noted between neonates and adults, but the impact on antivenom efficacy was not assessed [47,76,77,78]. The venom of the Pakistan locality of *Daboia russelii* varies between neonates and adults in a manner reflective of diet, with the neonate phenotype more potent on amphibian plasma reflective of amphibians being a higher proportion of their diet at this life stage [24]. In this case, there was no significant difference in antivenom efficacy, as there was no significant difference in effects upon mammalian plasma, including humans. Similarly, *Trimeresurus albolabris* neonate and adult venoms have the same level of potency in the pseudo-procoagulant action upon fibrinogen, with antivenom being equally effective against both [79]. However, for *Bothrops jararacussu,* while the venom biochemistry was similar between neonates and adults, the neonates were more potent and, therefore, compared to adults, the neonate venom required more antivenom to neutralize an equal mass of venom. [59]. A similar scenario was evident in a study on *Lachesis muta* venoms, where neonate venoms required over 50% more antivenom than adults to neutralize the same mass of venom [80]. In more extreme cases, if age-related venom variation targets different pathophysiological targets, this makes traditional antivenom selection based on snake species even more problematic, thus necessitating more consideration in antivenom manufacturing [33]. For example, the *Crotalus culminates* adult venom phenotype is an anticoagulant, but the neonate venom phenotype is a procoagulant [42]. As the regionally available antivenoms are made using anticoagulant pit viper venoms, the procoagulant neonate venom is not neutralized. Another example is the Australian brown snake species (*Pseudonaja* spp.), whereby juveniles are nocturnal lizard specialists that produce exclusively neurotoxic venoms, whereas diurnal adults produce venoms dominated by procoagulant toxins that effectively subdue mammalian prey [37,52]. In the case of *Crotalus molossus nigrescens,* the antivenom is unable to neutralize specific effects due to crotamine peptides [81].

*V. ammodytes* (Long-Nosed Viper) (Linnaeus, 1758) is widespread across southern Europe, and the subspecies *Vipera ammodytes meridionalis* (Eastern Long-Nosed Viper) (Boulenger, 1903) is restricted to Greece and Turkish Thrace [82,83]. This species contains a diverse array of toxins, which have a myriad of effects, including coagulotoxicity, myotoxicity, and neurotoxicity [84,85,86,87]. Traditionally considered the most dangerous snake in Europe because of the combination of potent venom and wide distribution overlapping with human population centers, *V. ammodytes* is a medically significant species capable of delivering a life-threatening bite [12,88,89]. Clinical records of *V. ammodytes* evenomations show it can cause symptoms such as pain, swelling, paralysis, and coagulopathy, which appear to be consistent with the proteomic composition of their venom [84,90,91]. The procoagulant action of *V. ammodytes* is primarily driven by the snake venom metalloproteases (SVMPs) found in the venom [92], with *V. ammodytes* the most procoagulant species of the *Vipera* genus [12]. It has also been shown that kallikrein-scaffold serine proteases, also present in the venom, are able to activate Factor X, but the concentration of this toxin type is very low [93]. As such, the contribution of kallikrein-scaffold serine protease enzymes to the overall procoagulant potency is marginal relative to that of the SVMPs. Consistent with this, metalloprotease inhibitors can restore clotting [12].

In Europe, bites from *V. ammodytes* are treated with Viperfav, a commercial antivenom prepared against European viper venoms [91,94]. However, when a shortage of Viperfav occurs, *V. ammodytes* envenomation is treated with ViperaTAb, an antivenom primarily used to treat *V. berus* bites. The efficacy of ViperaTAb against *V. ammodytes* has been reported to be limited, especially for severe symptoms [94,95]. A newly developed polyvalent antivenom, Inoserp Europe, was also reported as a possible treatment for *V. ammodytes* bites [12,94,96].

*V. ammodytes* effects upon clotting varies between subspecies [12]. It has also been shown that *V. a. meridionalis* is more complex and potent than that of the nominate subspecies, *V. a. ammodytes* [84]. However, no studies have investigated the possible ontogenetic variation in *V. ammodytes* venoms. To fill this knowledge gap, we compare neonate with adult venoms for their relative procoagulant potency and compare the impact upon the efficacy of three antivenoms (Viperfav, ViperaTAb, and Inoserp Europe).

## 2. Results

**Figure 1:**
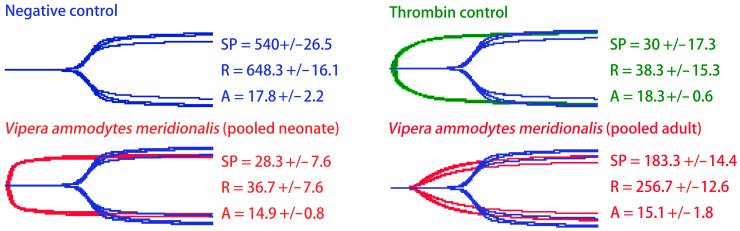
Thromboelastography using human plasma (1800 s total run time). Blue traces = spontaneous clot control (negative control), green traces = thrombin control, and red traces = venom samples. All traces are overlaid with the spontaneous clot control. SP = the split point, the time in seconds until clot formation begins. R = reaction time, the time in seconds until a detectable clot (>2 mm) is formed. A = amplitude, the width of tracing at the latest time point, representing clot strength (mm). Data are n = 4 mean ± standard deviation. Thrombin control is at a concentration of 1.94 NIH units/mL. Venom samples are at a concentration of 19.44 μg/mL.

Thromboelastography on human plasma (Figure 1) indicated both neonate and adult venom initiate clotting of plasma significantly faster than the spontaneous control (*p* < 0.001). However, there was a strong ontogenetic signal, with the neonate venom phenotype inducing clotting significantly faster than the adult (*p* < 0.001). 

**Figure 2:**
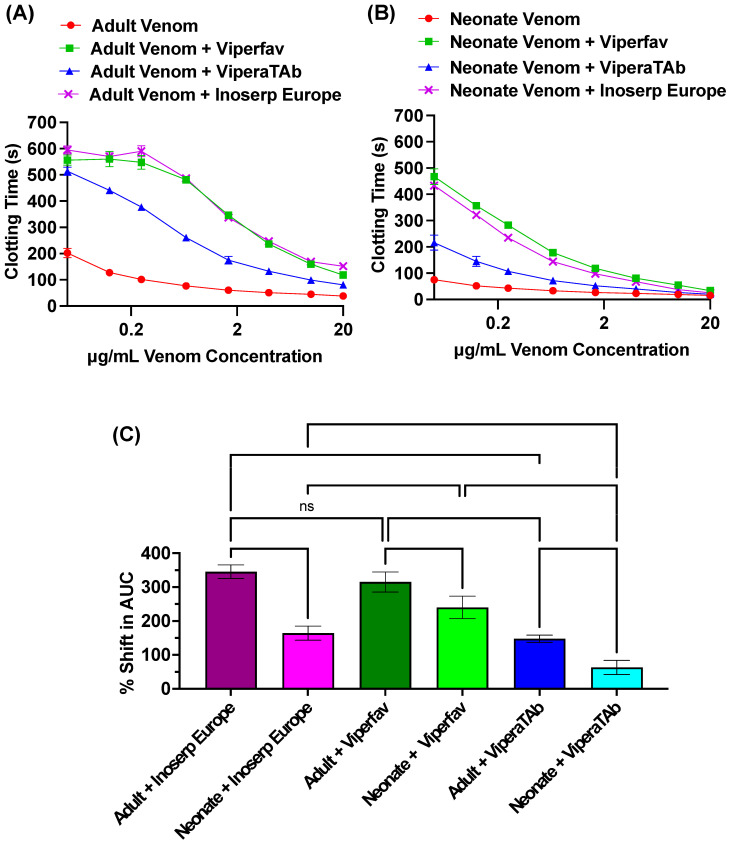
Logarithmic views of (**A**) adult and (**B**) neonate venom and antivenom plasma clotting dose-response curves (0.05, 0.125, 0.25, 0.66, 1.66, 4, 10, and 20 μg/mL). (**C**) Relative shifts in the area under the curve (AUC) for the venom and antivenom plasma clotting dose-response curves. No antivenom effect = 0%. *p*-values are comparisons between neonate and adult venoms within the same antivenom type, comparisons between antivenom types for neonate venom, and comparisons between antivenom types for adults. *p*-values classifications are as follows: ns = not significant (0.62 in this case). Statistics are Brown–Forsythe and Welch ANOVA tests with post-hoc Dunnett’s T3 multiple comparisons. All data are n = 3 ± standard deviation.

Subsequent Stago STA-R Max coagulation tests confirmed the thromboelastography results. At the maximum venom concentration tested (20 µg/mL), both neonate and adult venom significantly shortened clotting time (*p* < 0.0001) relative to the spontaneous clotting control of 407.6 ± 6.8 s with the adult clotting the plasma in 38.367 ± 2.12 s and neonate in 15.60 ± 0.61 s. The neonate venom was significantly faster than the adult (*p* = 0.001523), with the adult 246.46 ± 22.07% slower. Concentration–response curves (venom-only line graphs in Figure 2A,B) showed a similar pattern. Using the area under the curve (AUC) to compare potency shows the neonate venom was significantly more potent (AUC = 411.9 ± 33.94) than the adult venom (AUC = 964.3 ± 52.95) (*p* = 0.000299). Consistent with the adult venom type being used in antivenom production, antivenom testing revealed adult venom was better neutralized than neonate venom for all antivenoms tested (Figure 2). Differences in rank order of relative antivenom potency within each venom were as follows: adult Inoserp = Viperafav > ViperaTAb; and neonate Viperafav > Inoserp > ViperaTAb (Figure 2A–C).

**Figure 3:**
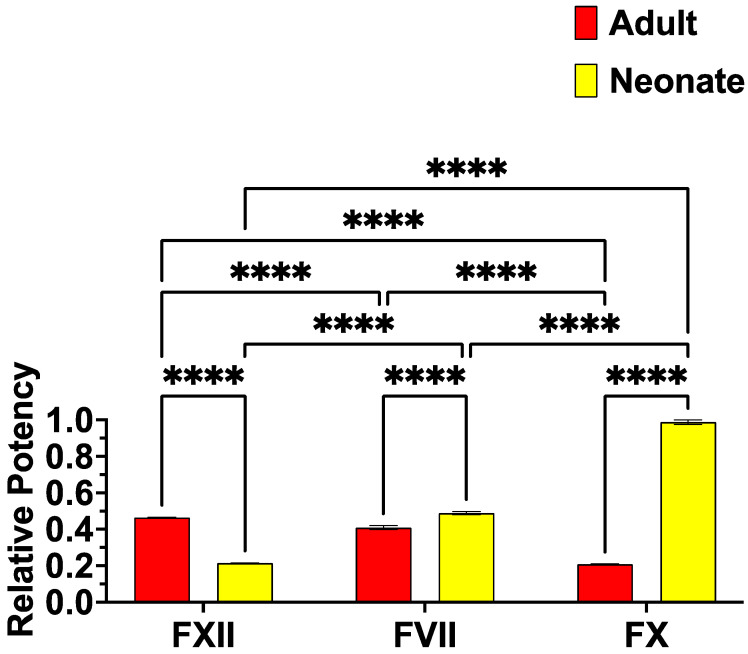
Adult and neonate relative ability to convert clotting factor zymogens into their corresponding activated enzyme. *p*-values are comparisons between neonate and adult venoms within the same factor type, comparisons between factor types for neonate venom, and comparisons between factor types for adults. *p*-values classifications are as follows: **** = *p* ≤ 0.0001. Statistics are Brown–Forsythe and Welch ANOVA tests with post-hoc Dunnett’s T3 multiple comparisons. Data are n = 3 mean ± standard deviation.

To ascertain the biochemical mechanisms responsible for the procoagulant toxicity upon plasma, tests were undertaken to determine which clotting factor zymogens were converted by the venoms into the activated enzymes. While Factors XI (FXI), FIX, and FII (prothrombin) were not activated, FVII, FX, and FXII were (Figure 3). Consistent with having a faster plasma clotting activity, the neonate venom was a much stronger FX activator than the adult and slightly more potent upon FVII. Conversely, the adult was more potent upon FXII than the neonate, activated FXII slightly more potently than FVII, and was least potent on FX.

## 3. Discussion

Our study found that while both neonate and adult *V. a. meridionalis* venom produced procoagulant actions on human plasma, significant ontogenetic variation in potency of effects between the two phenotypes was displayed (Figure 1 and Figure 2A,B). Results revealed neonate venom produces more potent procoagulant effects than adult venom. Venom ontogenetic shifts in *Vipera* species have been poorly studied, with this study being the first report of ontogenetic variation on *V. a. meridionalis* venom. Avella et al. showed an ontogenetic shift in the venom composition of *Vipera latastei*, a species closely related *to V. ammodytes*, with neonate venoms having a higher proportion of SVMPs than adults [47]. Consistent with a size-based variation in venom biochemistry, an examination of *V. monticola* subspecies that varies significantly in adult size revealed the subspecies with the smallest adult size (*V. m. atlantica*) had venoms with the highest SVMP content (13.2%), while the subspecies with the largest adult size (*V. m. saintgironsi*) had the lowest SVMP content (6.3%) [97]. However, it is important to note that neither study included functional assays, which is important as SVMPs are multifunctional. Consequently, neither study was able to inform about ontogenetic/size-related changes in procoagulant potency. As such, the current study is the first to investigate age-related variations in clotting factor action by *Vipera* venoms.

As diet an important selective force that shapes venom composition [37,65,98], prey specialization is the most likely major driver of ontogenetic venom variation on venomous snakes. The diet of *V. a. meridionalis* is reported to show ontogenetic variations, with juvenile vipers feeding on lizards and adults predominantly preying on birds and mammals [99,100,101]. The predatory ecology must also be considered, such as the extreme variation in the Australian elapid genus *Pseudonaja* (brown snakes)*,* whereby neonates are neurotoxic nocturnal specialists on sleeping lizards, while adults are procoagulotoxic diurnal pursuit predators of small mammals [37,52]. In contrast, the sister genus *Oxyuranus* does not display age-related venom effects, as they are diurnal pursuit predators of small mammals at all life stages [37]. Alternate theories have been proposed. One is that as juvenile snakes produce a limited amount of venom; they require stronger coagulopathic effects in venom to subjugate and kill prey [47]. However, a limitation of this theory is that while the smaller snakes produce less venom, they also feed on proportionally smaller prey. Another theory is that adults that feed upon larger prey may invest venom effects to facilitate consumption [102]. However, data to support this theory are lacking, and in fact, this theory has been proposed as invalid [103].

The ontogenetic shift in the diet of *V. latastei* has been reported to be similar to that of *V. ammodytes*, with juveniles of both species predominantly feeding on ectothermic prey and adults mainly predating on endotherms [47,99,104]. Paralleling this are juveniles with higher concentrations of SVMP enzymes [47], the toxin type responsible for procoagulant toxicity in this genus [12]. As such, the data in this are consistent with the ontogenetic variation in procoagulant effects produced by *V. a. meridionalis* venom is driven by a relative abundance of SVMP toxins. Therefore, this finding also provides a testable hypothesis for future research that *V. latastei* will show a similar ontogenetic variation in procoagulant potency.

The results of this study extend beyond biological theory and into the realm of human snakebite by providing data useful in the evidence-based design of clinical management strategies for the envenomed patient. Based on our results on antivenom efficacy, all three tested antivenoms showed higher efficacy against adult venom than neonate venom. However, in the treatment of an envenomation, this would, of course, be offset by the proportionally small venom yield of smaller specimens [105,106,107].

Viperfav, which is currently used to treat *V. ammodytes* bites [89,95], ranked as the most effective antivenom against neonate venom and the second most effective against adult venom on human plasma (Figure 2). The newly developed polyvalent antivenom Inoserp Europe also displayed effective neutralization against the coagulopathic effects caused by both neonate and adult *V. a. meridionalis* venom. This is consistent with previous results, which showed Inoserp Europe to be the most effective against the procoagulant effects of 12 *Vipera* species [12]. However, an in vivo mouse study identified ViperaTAb as more effective against *V. ammodytes* venoms from Croatia [94]. By contrast, ViperaTAb had limited effects on counteracting the procoagulant activity of both neonate and adult *V. a. meridionalis* venom in this study. This is not surprising as ViperaTab is immunized with only the venom of *V. berus*. Moreover, previous literature suggested limited effects of this antivenom against severe *V. a. meridionalis* envenomation [94,95] and poor performance compared to both Inoserp Europe and Viperfav in vitro [12].

Our study further interrogated the fundamental biochemistry underpinning the ontogenetic venom variation in *V. a. meridionalis*. Consistent with more potent procoagulant effects, neonate venom was a stronger activator of clotting factors, particularly FX (Figure 3). FX being the strongest activated zymogen is consistent with those of previous studies, which also showed potent FX activation by *V. ammodytes* and other species of *Vipera* [12,92,93]. However, this study was the first to show FVII or FXII activation for any *Vipera* venom. This adds to the growing body of literature regarding reptile venoms being able to activate diverse clotting factors besides just FII (prothrombin) or FX, including the following: *Oxyuranus* and *Pseudonaja* species (FVII activation in addition to FII) [52,108]; natricine species within the *Rhabdophis* genus (FVII >> FIX > FXII > FII > FX); and the viperid snake *Porthidium volcanicum* (FVII > FXII > FXI > FX) [15]; and *Heloderma* species of anguimorph lizards (FVII and FXII) [109].

An important caveat is that while our study provides evidence of the ontogenetic variation in coagulotoxic venom components of *V. a. meridionalis* and its impact on antivenom efficacy, possible ontogenetic shifts in other pathophysiological effects also need to be explored. Beyond the potent procoagulant components, neurotoxins and cardiotoxins, such as vipoxin and ammodytin L, are also present in *Vipera ammodytes* venom [12,84,88,110]. Envenomation, thus, can possibly result in vessel and myocardial dysfunction and cranial nerve paresis or paralysis [96]. Neurotoxicity of *V. ammodytes* is also relevant to antivenom efficacy. In the case of antivenom ViperaTAb, while shown to have some effects against coagulopathic venom in the present study, it was reported to have no effects at all on neurological signs caused by *V. ammodytes* bite [95]. Exploring ontogenetic shifts in these other pathophysiological actions and the impact on antivenom efficacy will enable us to fully understand the potential clinical effects of *V. a. meridionalis* envenomations, as well as the evolutionary influences underlying it.

## 4. Materials and Methods

### 4.1. Venom

Venom work was conducted under the University of Queensland Animal Ethics Approval 2021/AE000075 and UQ Biosafety Committee Approval # IBC/134B/SBS/2015. Six lyophilized *Vipera ammodytes meridionalis* venoms were provided by alpha-biotoxins. Samples included venom from two wild adult individuals (male and female, both from Peloponese Greece) and their offspring (five neonates, sex unknown, milked at 3 months of age). Venom samples were stored in a −80 °C freezer until use. Venom stocks were reconstituted to a 1mg/mL working stock with a 50% double deionized water and 50% glycerol mix to preserve enzymatic activity. Concentrations of venom samples were determined by a Thermo Fisher Scientific NanoDrop 2000 UV–Vis Spectrophotometer (Thermofisher, Sydney, NSW, Australia). Prepared venom stocks were stored in a −20 °C freezer.

### 4.2. Plasma Coagulation Assay Approvals

Human-plasma work was performed under University of Queensland Biosafety Approval #IBC134BSBS2015 and Human Ethics Approval #2016000256. Australian Red Cross (44 Musk Street, Kelvin Grove, QLD 4059, Australia) supplied human platelet-poor plasma (3.2% citrated) under research approval #16- 04QLD-10. Samples were flash-frozen in liquid nitrogen and stored in 1.5 ml aliquots at −80 °C until required. For testing, plasma was defrosted in a 37 °C water bath for 5 min before use.

### 4.3. Thromboelastography

A Thrombelastograph 5000 Haemostasis analyzer (Haemonetics, Haemonetics Australia Pty Ltd., North Rdye, Sydney, Australia) was employed to measure the effect of *V. a. meridionalis* venom on human-plasma clot strength, “TEG^®^ 5000 disposable cups and pins clear” were used (Haemonetics^®^, REF 6211). In each assay, 72 μL 0.025M CaCl_2_ (Stago Cat# 00367), 72 μL phospholipid (Stago Cat# 00597) solubilized in Owren Koller (OK), and 20 μL OK buffer (Stago Cat# 00360) were pipetted into cups, followed by 7 μL 50% deionized water/50% glycerol for the spontaneous clot control, 7 μL of thrombin (Stago Cat#00673 Liquid Fib, thrombin concentration of 100 NIH units/mL) for the thrombin control and 7 μL of 1 mg/mL venom stock for the clot strength assays. After all reagents were added, 189 μL human plasma (thawed for 5 min in a 37 °C water bath) was pipetted into cups. Testing was conducted at 37 °C. Each assay was performed for 30 min. Traces were exported from the analyzer and processed in Adobe Photoshop to create figures.

### 4.4. Coagulation Curves

The ability of venoms to clot human plasma at different concentrations was measured with a Stago STA-R Max hemostasis analyzer (Stago, Asnières sur Seine, France). Plasma samples were thawed in a 37 °C water bath for 5 min prior to testing. The clotting time of each venom sample was measured in triplicate at eight different concentrations (20 μg/mL, 10 μg/mL, 4 μg/mL, 1.6 μg/mL, 0.66 μg/mL, 0.25 μg/mL, 0.125 μg/mL, and 0.05 μg/mL). For testing, 1 mg/mL venom stock was diluted with OK buffer to 0.1 mg/mL and placed into the analyzer. For the 20 μg/mL concentration, 50 μL 0.025 M CaCl_2_, 50 μL phospholipid solubilized in 25 μL OK buffer, and 50 μL of 0.1 mg/mL venom were automatically pipetted into a cuvette and incubated for 120 s at 37 °C. Following incubation, 75 μL of human plasma was added to the cuvette, and clotting time was measured using a mechanical, viscosity-based system. For additional concentrations, the volumes of venom and OK buffer added to the cuvette were adjusted. The final cuvette volume for all concentrations was 250 μL.

### 4.5. Antivenom Neutralization Studies

Antivenom assays were also performed on a Stago STA-R Max hemostasis analyzer to test the efficacy of antivenom in neutralizing the coagulotoxic activity of *V. a. meridionalis* venom. The antivenoms tested were Inoserp Europe (lot # 9IT03006), a 22.5 mg/mL F(ab′)2 antivenom made using an immunizing mixture consisting of *Macrovipera lebetina cernovi*, *M. l. obtusa*, *M. l. turanica*, *M. schweizeri*, *Montivipera xanthina*, *Vipera ammodytes*, *V. aspis*, *V. berus*, and *V. latastei*; MicroPharm VIPERFAV (lot #P4A281V), a 100 mg/mL F(ab′)2 antivenom made using an immunizing mixture consisting of *Vipera ammodytes*, *V. aspis*, and *V. berus*; and MicroPharm ViperaTAb (lot #VPT 002000), a 24.6 mg/mL Fab antivenom made using *V. berus* as the sole venom in the immunizing mixture.

Antivenoms were diluted with OK buffer to a concentration of 5%. The same procedure as in plasma coagulation assays (Section 4.4) was followed, except 25 μL of OK buffer was replaced with 25 μL of 5% antivenom, leading to a final cuvette concentration of 0.5%.

### 4.6. Clotting Factor Activation Assays

Clotting factor activation assays were performed with Fluoroskan Ascent (Thermo Scientific, Vantaa, Finland) to detect clotting factor (Factor VII, X, XI, XII, and prothrombin) activation and compare the relative ability of factor activation between neonate and adult *V. a. meridionalis* venom. Reaction stoichiometry and reaction conditions were as per [109]. Reagents were automatically plated in 384-well plates (black, lot#1171125; Nunc Thermo Scientific, Rochester, NY, USA) by a Hamilton Vantage Liquid Handling System (USA). Plates were manually loaded into the Fluoroskan Ascent, and measurement started. The Fluoroskan Ascent automatically pipetted 70 μL of buffer, which contained 5 mM CaCl_2_, 150 mM NaCl, 50 mM Tris-HCl (pH 7.3) and Fluorogenic Peptide Substrate (ES011Boc-Val-Pro-Arg-AMC. Boc: t-Butyloxycarbonyl; 7-Amino-4-methylcoumarin; R & D systems, Cat# ES011, Minneapolis, MN, USA) in a 500:1 ratio, to each well to start the reaction. The plate was warmed up at 37 °C and shaken for 3 s in Fluoroskan Ascent before each measurement. The reaction was carried out 300 times at 390 (excitation)/460 nm (emission), and the fluorescence generated by the cleavage of the substrate was measured by Ascent Software v2.6 (Thermo Scientific, Vantaa, Finland) every 10 s. To obtain final results, subtraction of “venom without zymogen” values from “venom with zymogen” values was performed, which nullified artificial increments of the fluorescence values caused by venoms that work directly on the substrate. Finally, the results from the subtractions were normalized as a percentage relative to the positive control (activated factors/enzyme (note: FXII was activated by using Kaolin and that solution used as control)) by processing in Excel and then analyzing in GraphPad PRISM 8.1.1 (GraphPad Prism Inc., La Jolla, CA, USA).

### 4.7. Statistical Analyses

GraphPad PRISM 8.1.1 (GraphPad Prism Inc., La Jolla, CA, USA) was used to perform statistical analyses. For the plasma clotting time of venom and venom incubated with antivenom, an area under the curve (AUC) was generated based on venom curves. To test and compare antivenom efficacy, an X-fold shift was calculated with the following formula:

$$X\;\mathrm{fold}\;\mathrm{shift}=\frac{\mathrm{AUC}\;\mathrm{of}\;\left(\mathrm{venom}+\mathrm{antivenom}\right)}{\mathrm{AUC}\;\mathrm{of}\;\mathrm{venom}}-1$$

The value of the X-fold shift indicates the neutralization of venom activity achieved by antivenom. An AX-fold shift of 0 indicates no neutralization, while a value above 0 indicates neutralization. These values were converted to a percent by multiplying by 100. The statistically significant results in percent AUC shift were classed as *p* < 0.05.

## Data Availability

All data are presented in the figures.
